# The Impact of Confluence Types of the Right Gastroepiploic Vein on No. 6 Lymphadenectomy During Laparoscopic Radical Gastrectomy

**DOI:** 10.1097/MD.0000000000001383

**Published:** 2015-08-21

**Authors:** Long-Long Cao, Chang-Ming Huang, Jun Lu, Chao-Hui Zheng, Ping Li, Jian-Wei Xie, Jia-Bin Wang, Jian-Xian Lin, Qi-Yue Chen, Mi Lin, Ru-Hong Tu

**Affiliations:** From the Department of Gastric Surgery, Fujian Medical University Union Hospital, Fuzhou, Fujian Province, People's Republic of China

## Abstract

This study investigated anatomical variations in the confluence types of the right gastroepiploic vein (RGEV) to improve knowledge regarding no. 6 lymphadenectomy for laparoscopic gastrectomy.

The RGEV drainage patterns of 144 patients who were diagnosed with gastric cancer and underwent laparoscopic distal gastrectomy at our department from July 2010 to June 2011 were prospectively collected and retrospectively analyzed, and we compared the impact of different drainage patterns on no. 6 lymphadenectomy.

The RGEV confluence types were classified into 6 categories in this study. Types I, II, and III, which were observed in 53 (36.8%), 27 (18.8%), and 21 (14.6%) cases, respectively, were the most frequently found during gastrectomy. All 3 of these types included a gastropancreatic trunk and were defined as the gastropancreatic group (GP group). In addition, 15 cases (10.4%) were categorized as type IV, 19 (13.2%) were categorized as type V, and 9 (6.3%) were categorized as type VI. These 3 types, which could form a gastrocolic trunk, were defined as the gastrocolic group (GC group). No significant differences were found with respect to the clinicopathological characteristics, postoperative morbidity, perioperative mortality, and 3-year overall survival rates after surgery between the 2 groups (all *P* > 0.05). However, the mean no. 6 lymph node (No. 6 LN) dissection time, the mean blood loss due to No. 6 LN dissection and the rate of infrapyloric vascular injury were significantly increased in the GC group compared with the GP group (all *P* < 0.05).

The RGEV exhibits 6 types of drainage patterns, and the division points of this vein during laparoscopic gastrectomy depend on the different drainage patterns. For types IV, V, and VI, the surgeon should carefully vascularize and divide the RGEV above its confluences during surgery.

## INTRODUCTION

Lymph nodes (LNs) located in the infrapyloric area in front of the pancreatic head are also referred to as No. 6 LNs. The No. 6 LN area has been defined by the Japanese Gastric Cancer Association (JGCA)^1^ as the area along the first branch of the right gastroepiploic artery (RGEA) down to the confluence of the right gastroepiploic vein (RGEV) and the anterior superior pancreaticoduodenal vein (ASPDV). Based on the categorization of the JGCA, some Japanese researchers^2,3^ have divided the No. 6 LNs into 3 sections, including the areas along the proximal portions of the RGEA (No. 6a), RGEV (No. 6v), and infrapyloric artery (IPA) (No. 6i).

The frequency of No. 6 lymph node metastasis (LNM) varies between 26.0% and 34.0%. In advanced middle and lower gastric cancers in particular, the incidence of No. 6 LNM varies from 34.2% to 41.0%,^4–6^ which is the highest incidence of LNM among all groups. Therefore, the No. 6 LNs should be removed during radical gastrectomy.^7–9^ Shinohara et al^2^ have described a new laparoscopic technique for dissection of No. 6 LNs in gastric cancer surgery that follows topographic anatomical logic and the venous confluence of the RGEV and ASPDV, which is defined as the lower border of the No. 6 LNs by the JGCA and is the starting point of No. 6 lymphadenectomy during laparoscopic surgery. However, the RGEV exhibits numerous drainage patterns under the area at the pancreatic head, which is an important and complicated anatomical area for gastric cancer surgery.^10,11^ Therefore, anatomical knowledge of the confluence of the RGEV is necessary not only for surgical procedures but also for oncologically reliable No. 6 LN dissection.

Although the anatomy of the gastrocolic trunk of Henle (GTH) and the RGEV have been explored in numerous studies using open surgery or radiological technology in the past years,^12–14^ few studies have described the confluence of the RGEV using laparoscopic techniques due to its complexity and potential for hemorrhage during surgery. The laparoscopic anatomy of the RGEV is increasing in importance with the gradual popularization of laparoscopic radical gastrectomy. In the present study, we describe the confluence types of the RGEV in 144 patients who underwent laparoscopic radical gastrectomy and summarize the laparoscopic technique for the lower border of the No. 6 LNs during lymphadenectomy.

## MATERIALS AND METHODS

### General Conditions

This study included 144 patients who underwent laparoscopic distal gastrectomy at Fujian Medical University Union Hospital between July 2010 and June 2011. All of the patients underwent standard No. 6 lymphadenectomy based on Japanese gastric Cancer Treatment Guidelines (2010).^15^ The patients were found to have 6 types of RGEV confluence, including types I, II, and III, which were classified into the gastropancreatic group (GP group), and types IV, V, and VI, which were classified into the gastrocolic group (GC group). The clinicopathological characteristics, short-term curative effects, and long-term survival rates after surgery were retrospectively analyzed in the 2 groups. The ethics committee of the Fujian Union Hospital approved this retrospective study. Written consent was obtained from the patients for their information to be stored in the hospital database and used for research.

**FIGURE 1:**
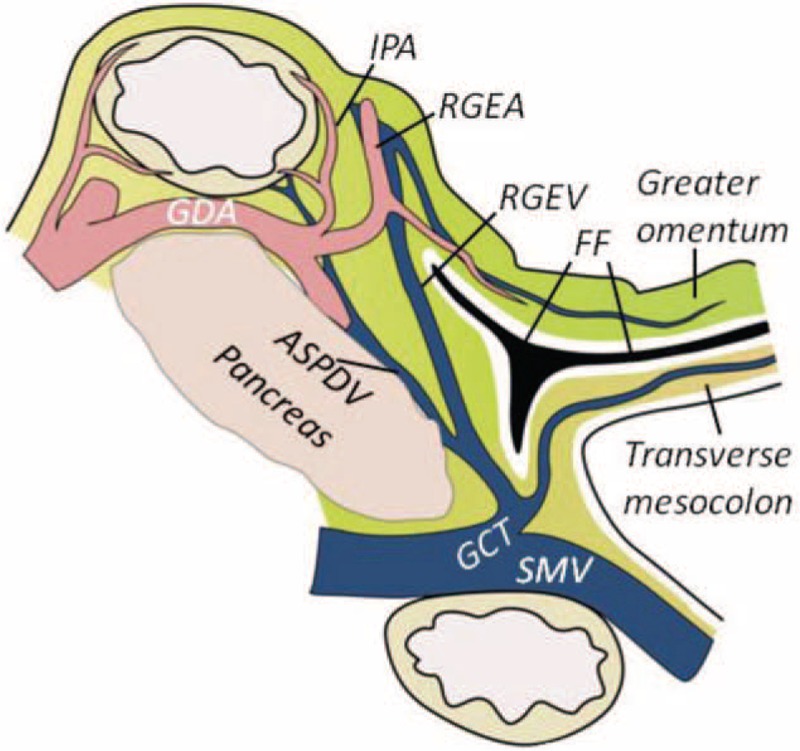
A sagittal section of the infrapyloric area. No. 6 LNs lie between the pancreatic head and the fusion fascia (FF). The upper border is located at the first branch of the RGEA, and the lower border is located at the confluence of the RGEV and ASPDV. ASPDV = anterior superior pancreaticoduodenal vein; GDA = gastroduodenal artery; GCT = gastrocolic trunk; IPA = infrapyloric artery; LN = lymph node; RGEA = right gastroepiploic artery; RGEV = right gastroepiploic vein; SMV = superior mesenteric vein.

Anatomical border of the infrapyloric No. 6 LN region: Based on the 3rd edition of the Japanese classification of gastric carcinoma (JCGC),^1^ the upper border of the No. 6 LN region was defined as the first branch and the proximal part of the RGEA, and the lower border included the confluence of the RGEV and ASPDV. Shinohara et al^2,3^ have described this region more in detail, stating that the No. 6 LNs lie between the pancreatic head and the fusion fascia (FF), which is termed the gastroduodenal ligament. The left side of the No. 6 LN area is located at the omental bursa, the right side is located at the gastroduodenal wall, the upper border is located at the first branch of RGEA, which supplies the greater curvature, and the lower border is situated at the confluence of the RGEV and ASPDV, which frequently exhibits anatomical variation during laparoscopic gastric cancer surgery (Figure 1). Estimated blood loss was based both on the contents of the suction container after adjusting for the irrigation used and on the surgeon's estimation using blood-soaked gauze. The No. 6 LN dissection time was defined as the time from exposure of the FF to the time that the lymphatic and fatty tissues in the infrapyloric area were dissected and removed en bloc. Postoperative morbidities were classified based on the Clavien–Dindo classification,^16^ and Grade I or II complications were defined as minor complications, whereas Grade III or higher complications were defined as severe complications. The staging of gastric tumors and LNs were performed according to the JCGC.^1^

### Surgical Technique

**FIGURE 2:**
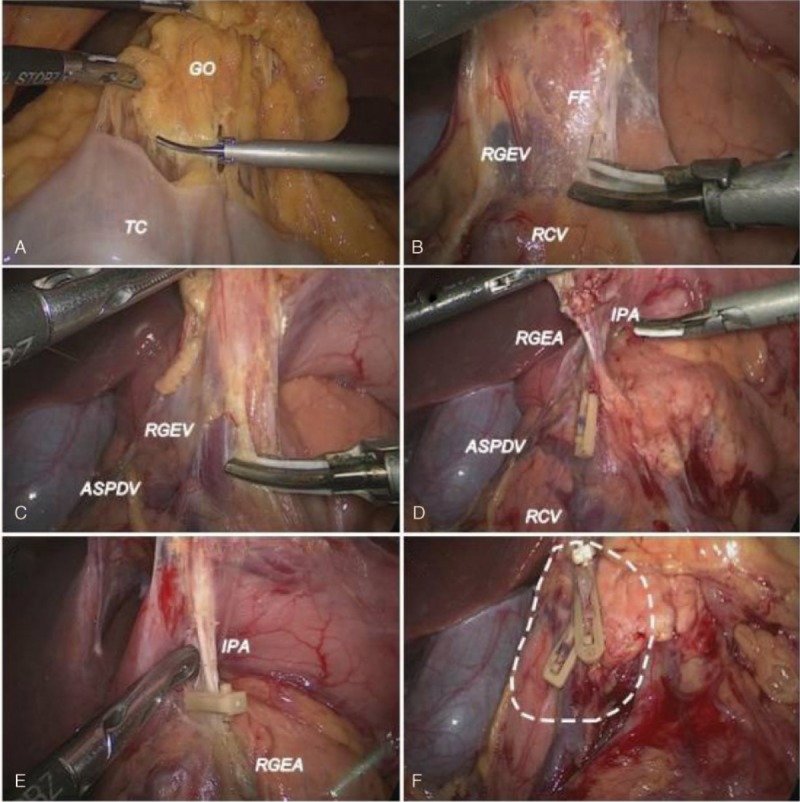
Surgical procedure of No. 6 lymphadenectomy. A: The greater omentum (GO) was repositioned above the transverse colon and divided within the avascular area near the midpoint to the hepatic flexure of the colon. B: Exposure of the fusion fascia (FF) on the frontal surface of the mesoduodenum to identify the right gastroepiploic vein (RGEV) and the confluence with the ASPDV under the FF. C: The FF was transected, and adipose tissue surrounding the RGEV was dissected to expose the RGEV, which then was divided with clamps above the confluence. D: The root of the RGEA and the infrapyloric artery (IPA) was exposed along the groove between the pancreatic head and the duodenum. E: The RGEA and IPA were divided at the roots. F: After removal of the No. 6 LN region. The broken line indicates the dissection margin. LN = lymph node.

Exposure of the FF: The greater omentum (GO) was repositioned above the transverse colon and the anterior wall of the stomach and divided in the avascular area, starting from the superior border of the transverse colon near the midpoint (Figure 2A). The division continued rightward until the hepatic flexure of the colon was reached. The divided omentum was then detached from the FF (also called the gastrocolic fascia), and the transverse mesocolon was also repositioned to identify the RGEV and the confluence with the ASPDV under the FF (Figure 2B).

Venous area of the No. 6 lymphadenectomy: The FF was transected, and adipose tissue surrounding the RGEV to the right of the inner border of the descending part of the duodenum up to the superior border of the pancreatic head was dissected to expose the RGEV (Figure 2C). After the RGEV was vascularized, it was divided by placing clamps above the confluence (Figure 2D).

Arterial area of No. 6 lymphadenectomy: After lifting the posterior wall of the gastric antrum and pressing down on the pancreas to expose the groove between the pancreatic head and duodenum, the gastroduodenal artery (GDA) was exposed and separated, and the root of the RGEA was also exposed along the terminal segment of the GDA (Figure 2D). The fatty lymphatic tissue around the RGEA was then dissected, and the root of the artery was vascularized and divided (Figure 2E). The IPA, which arises from the GDA, was divided as well. During dissection, injury to the IPA was carefully avoided to prevent bleeding. Finally, dissection was continued along the duodenum from the division of the RGEA to the pylorus. The lymphatic and fatty tissues, including the No. 6 LNs in the infrapyloric area, were dissected and removed en bloc (Figure 2F).

### Postoperative Follow-Up

The patients were followed up after surgery by telephone calls, outpatient visits, and letters. The overall survival time was calculated from the date of diagnosis until the date of last contact, date of death, or date on which the survival information was collected. All surviving patients were followed up for more than 3 years.

### Statistical Analysis

All measurement data were presented as the mean ± SE and analyzed using SPSS 17.0 for Windows (SPSS, Chicago, IL). The data were compared using the chi-square test, Fisher's exact test, or unpaired Student *t* test, as appropriate. A *P* < 0.05 was considered statistically significant.

## RESULTS

### Confluence Type of the RGEV

**FIGURE 3:**
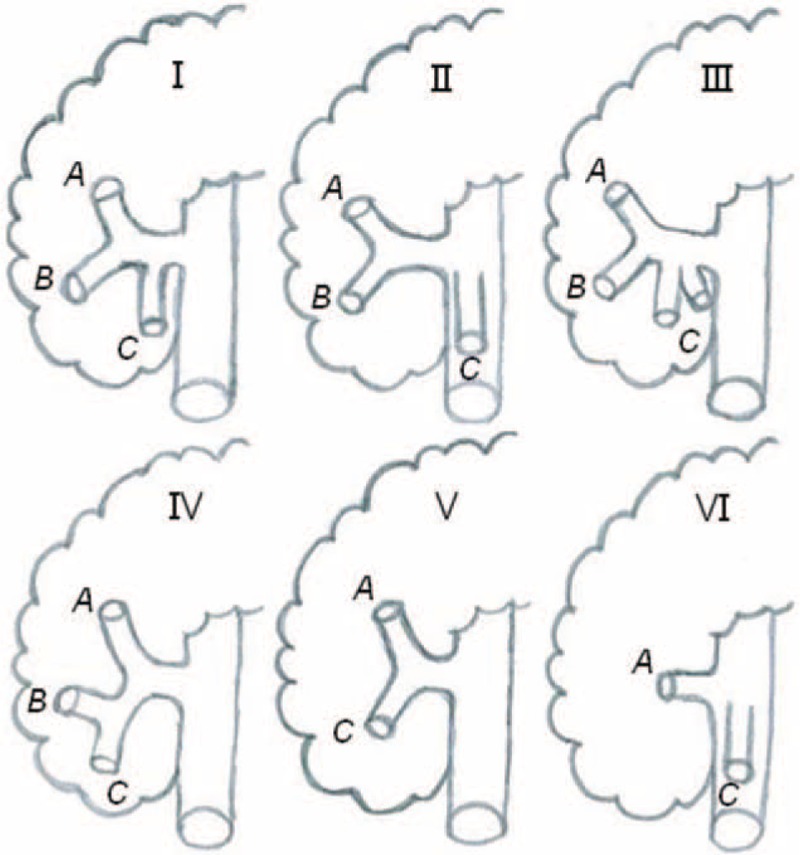
Schematic diagrams of the 6 types of confluence of the right gastroepiploic vein (RGEV) and surrounding veins. A: The RGEV. B: The anterior superior pancreaticoduodenal vein (ASPDV). C: A superior right colic vein (SRCV) or a right colic vein (RCV).

**FIGURE 4:**
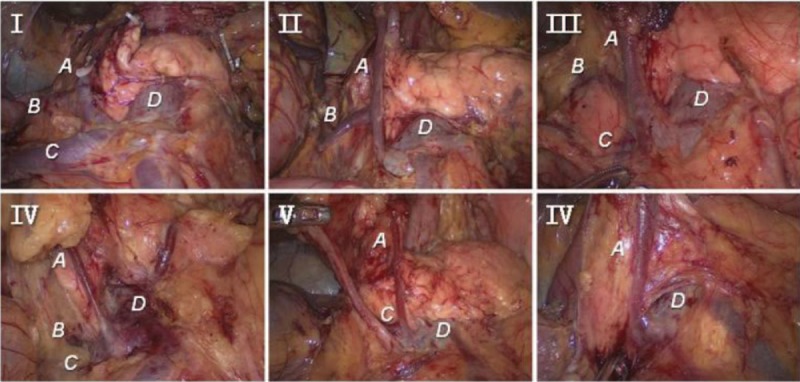
Representative surgical pictures of the 6 types of confluence of the right gastroepiploic vein (RGEV) and surrounding veins. A: The RGEV. B: The anterior superior pancreaticoduodenal vein (ASPDV). C: A superior right colic vein (SRCV) or a right colic vein (RCV). D: The superior mesenteric vein (SMV).

The RGEV was present in all 144 patients, and the root was exposed and carefully ligated during surgery. Our study summarized the following 6 types of confluence among the RGEV and surrounding veins (Figures 3 and 4): type I (confluence with the ASPDV, with participation of a colic vein); type II (confluence with the ASPDV, without participation of a colic vein); type III (confluence with the ASPDV, with participation of a superior right colic vein [SRCV] and a right colic vein [RCV]); type IV (confluence with the co-trunk of the ASPDV and a colic vein); type V (confluence with a colic vein, without participation of the ASPDV); and type VI (separate termination of the RGEV in the superior mesenteric vein [SMV]). Fifty-three cases (36.8%) were categorized as type I, 27 (18.8%) were categorized as type II, and 21 (14.6%) were categorized as type III. A gastropancreatic trunk comprising the RGEV and ASPDV was observed in these 3 types, which were defined as the GP group. Fifteen cases (10.4%) were classified as type IV, 19 (13.2%) were classified as type V, and 9 (6.3%) were classified as type VI; these 3 types could form a gastrocolic trunk (GCT) and were defined as the GC group.

### Patient Clinicopathological Characteristics

This series included 108 men (75%) and 36 women with a mean age of 61.1 years (ranging from 31 to 88 years). Gender, age, body mass index, tumor size, tumor location, tumor differentiation, the depth of invasion, LNM, and the TNM stage did not differ between the 2 groups (all *P* > 0.05) (Table 1).

**TABLE 1:**
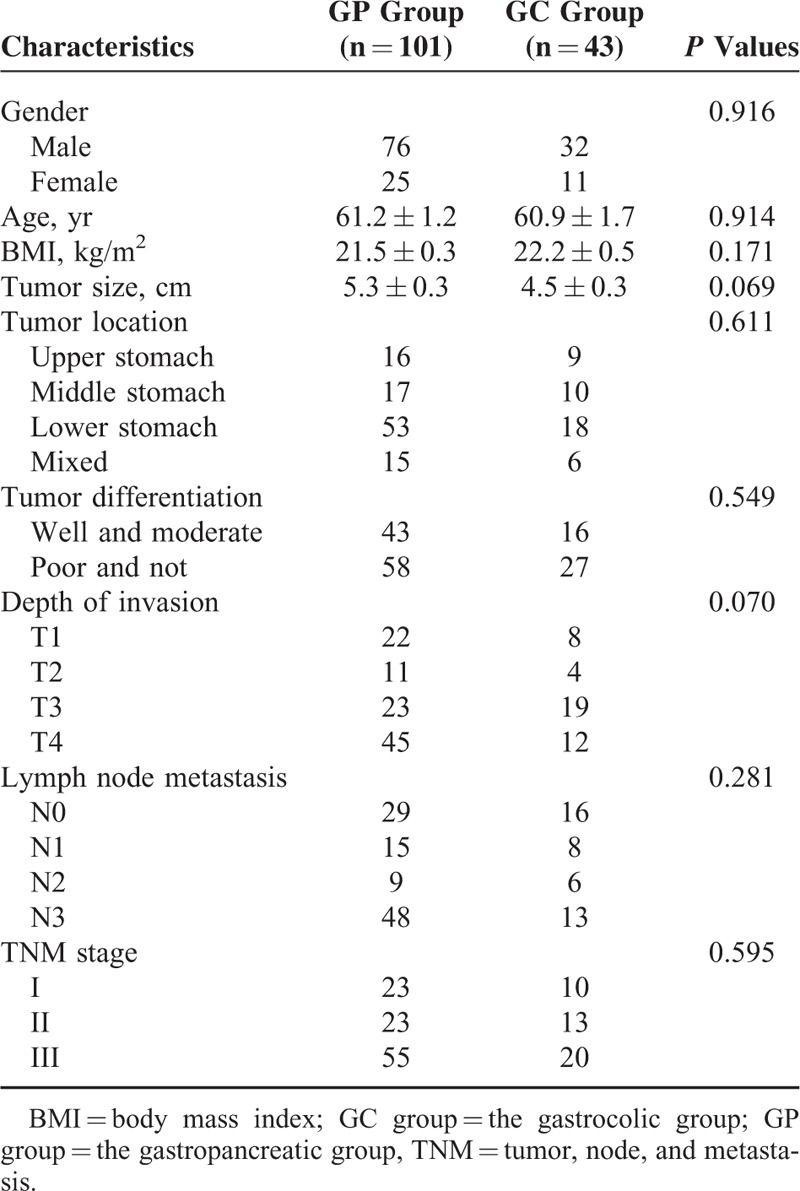
Comparison of Clinicopathological Characteristics Between the 2 Groups

### Intraoperative and Postoperative Characteristics

The surgical time, mean total blood loss, use of intraoperative and postoperative transfusions, mean number of harvested total LNs, mean number of harvested No. 6 LNs, lymph node metastasis rate (LMR), and ratio of metastatic lymph nodes (RMLs) in the No. 6 area were similar between the 2 groups (each *P* > 0.05). However, the mean No. 6 LN dissection time, the mean blood loss due to No. 6 LN dissection, and the rate of infrapyloric vascular injury were significantly increased in the GC group compared with the GP group (all *P* < 0.05). The times to first flatus, fluid diet, and soft diet and the length of hospital stay did not differ between the 2 groups (all *P* > 0.05) (Table 2).

**TABLE 2:**
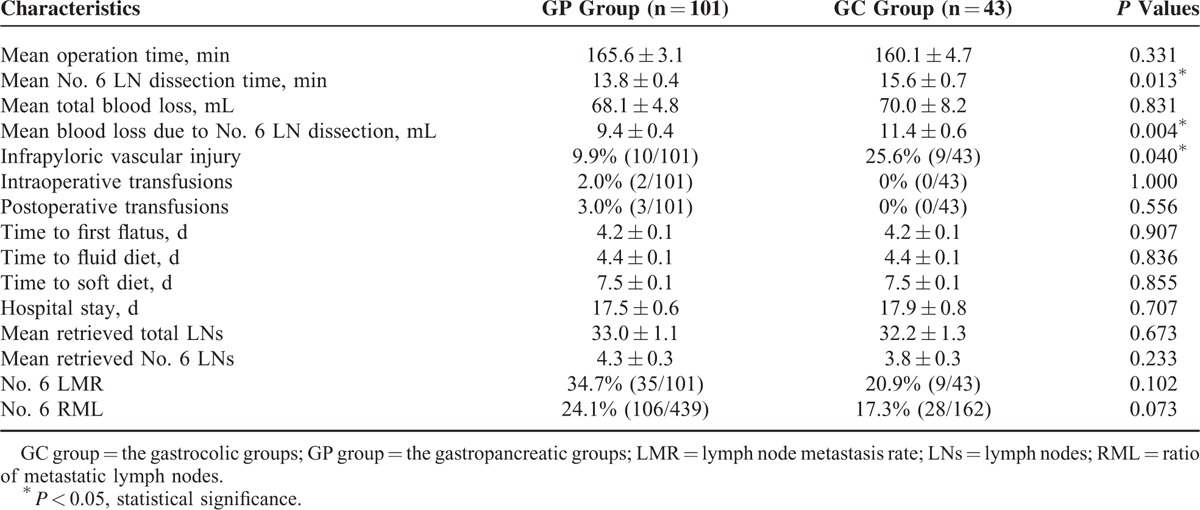
Intraoperative and Postoperative Characteristics Between the 2 Groups

### Morbidity and Mortality

The overall postoperative morbidity was 11.8% (17/144), with rates of 11.9% (12/101) in the GP group and 11.6% (5/43) in the GC group, which did not significantly differ (*P* > 0.05). Furthermore, the incidence of severe complications was compared between the 2 groups (GP group, 2.0% [2/101]; GC group, 0 [0/43]), and no difference was found (*P* > 0.05). The 30-day mortality rate in all patients was 1.4%, with rates of 1.0% (1/101) in the GP group and 2.3% (1/43) in the GC group, which did not significantly differ (*P* > 0.05) (Table 3).

**TABLE 3:**
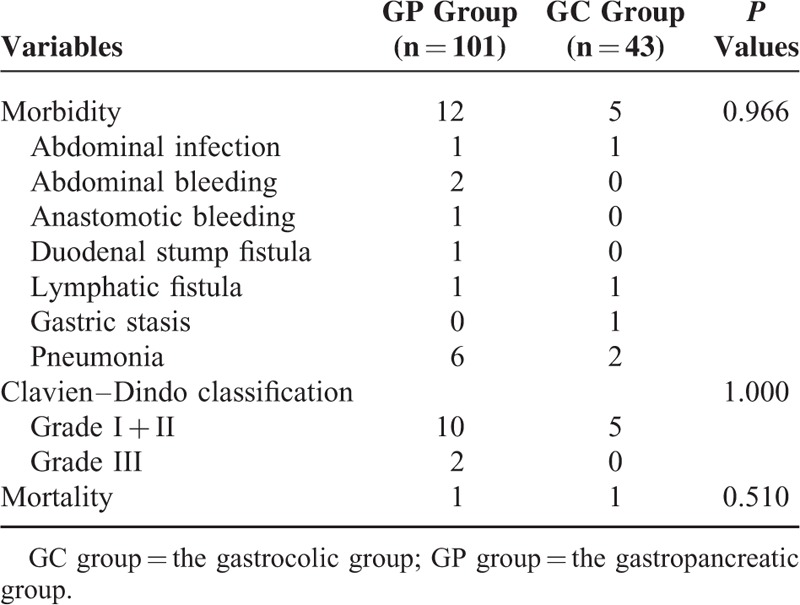
Comparison of Morbidity and Mortality Between the 2 Groups

There were 6 cases of pneumonia, 2 cases of abdominal bleeding, 1 case of abdominal infection, 1 case of anastomotic bleeding, 1 case of duodenal stump fistula, and 1 case of lymphatic fistula in the GP group, whereas there were 2 cases of pneumonia, 1 case of abdominal infection, 1 case of lymphatic fistula, and 1 case of gastric stasis in the GC group. The patient who was diagnosed with anastomotic bleeding and 1 of the 2 patients who were diagnosed with abdominal bleeding required a second operation, and all of the other postoperative complications were successfully treated by conservative methods. None of the patients died during hospitalization (Table 3).

### Postoperative Follow-Up

**FIGURE 5:**
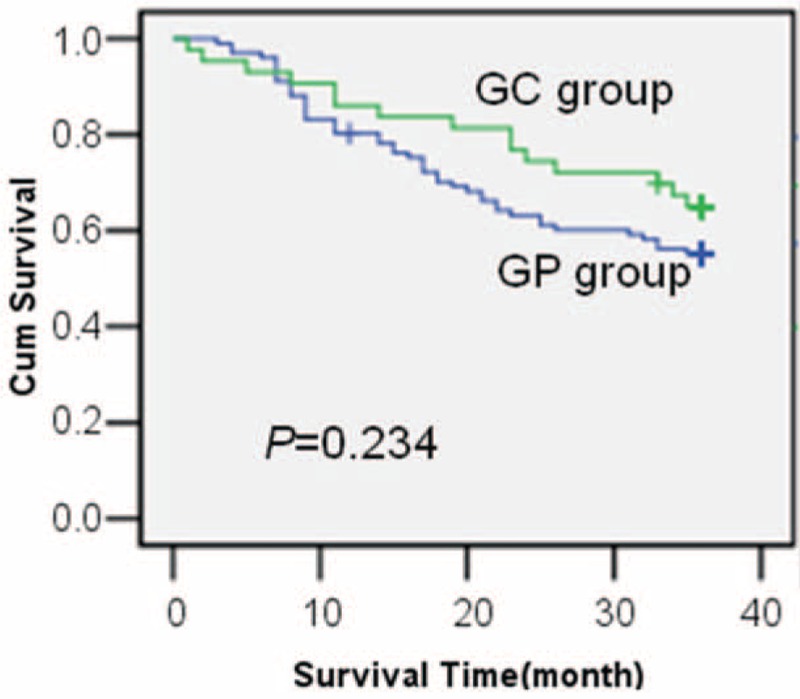
Kaplan–Meier curves for the patients in the GP and GC groups. The 3-year overall survival rates between the 2 groups were not significantly different (*P* = 0.234, log-rank test). GC group = the gastrocolic group; GP group = the gastropancreatic group.

Follow-up was conducted for all patients. We analyzed the survival time of the patients using Kaplan–Meier survival analysis. The results revealed that the median survival time among all patients was 36 months (ranging from 1 to 51 months). The 3-year overall survival rates in the GP and GC groups were 55.1% and 65.0%, respectively, and they did not significantly differ (*P* > 0.05) (Figure 5).

## DISCUSSION

Among the regional LNs of the stomach, the No. 6 LNs are located in an anatomically significant area. Infrapyloric LNMs occur frequently in advanced middle and lower gastric cancer^7^; therefore, these nodes should be removed during gastrectomy.^15^ The JGCA has defined the confluence of the RGEV and ASPDV as part of the efferent section of the No. 6 LN area.^1^ However, many complex anatomical variations of the RGEV have been revealed during surgery. Thus, we focused on the anatomy of this vein and the associated lymphadenectomy in this study.

The RGEV, which is responsible for drainage of the right part of the greater curvature, was initially described as 1 of the 2 veins forming the GCT by Henle in 1868.^17^ The ASPDV was added to the confluence as a third element by Descomps and De Lalaubie in 1962, and the trunk was then considered a tripod.^18^ Clinically, the anatomy of the venous tributaries of the RGEV under the infrapyloric area and at the anterior part of the pancreatic head is of interest because these veins must be carefully identified and dissected to avoid hemorrhages during not only gastrectomy but also certain pancreatic operations (Whipple resection, pancreatectomy).^19,20^ However, studies evaluating the confluence types of the RGEV and their incidence rates are rare in the literature, especially for laparoscopic gastrectomy. Jin et al^21^ summarized 4 types of RGEV confluence in 9 patients, including RGEV convergence with the ASPDV with no colic vein involvement in 11% of patients, the involvement of 1 colic vein in 33% of patients, the involvement of 2 colic veins in 45% of patients, and the involvement of 3 colic veins in 11% of patients. Lange et al^22^ reported 4 types of RGEV confluence in 37 patients, including RGEV confluence with only the ASPDV in 43% of patients, confluence with the ASPDV and then the colic vein in 38% of patients, separate termination in the SMV in 11% of patients, and confluence with a colic vein in only 8% of patients. However, these studies may not comprehensively summarize the confluence types of the RGEV due to their small sample sizes. In the present study, we carefully reviewed the venous anatomy of 144 patients and identified 2 groups and 6 types of confluence among the RGEV and its surrounding veins. To the best of our knowledge, the sample size in the present study is larger than that in other studies that have examined RGEV anatomy.

Most of the patients in this study belonged to the GP group (including types I, II, and III), in which the RGEV directly converges with the ASPDV, and the confluence occurs at the lower border of the No. 6 LNs. During No. 6 lymphadenectomy, the confluence between the RGEV and ASPDV should be vascularized first; the RGEV should then be divided above the confluence, and the dissection should begin with the confluence and continue upward until en bloc resection is achieved. However, only 29.8% of the patients belonged to the GC group (including types IV, V, and VI). For types IV and V, the RGEV directly converges with a colic vein (SRCV or RCV). During lymphadenectomy for these 2 types, the surgeon should carefully vascularize the confluence of the RGEV and the colic vein due to its complicated nature, divide the RGEV above the confluence and continue to dissect upward from it. Because the RGEV terminates separately in the SMV in type VI, the surgeon should entirely expose the SMV after confirming that no colic veins are joined and then divide the RGEV above the confluence and continue to dissect upward until achieving en bloc infrapyloric lymphadenectomy.

The RGEV is one of the most important blood vessels and requires careful exposure and division during gastric surgery. The confluence of the RGEV at the inferior border of the pancreas should be exposed carefully. If any vein is injured and bleeds during surgery, the anatomical layers will be unclear, which will increase the difficulty of lymphadenectomy, thereby affecting the surgical procedure, prolonging the operation time and increasing the complication rate. Thus, alterations in RGEV drainage patterns can affect the surgical process. In the present study, we observed that the surgical procedures for No. 6 lymphadenectomy required more time and resulted in increased infrapyloric vascular injury and blood loss in the patients in the GC group compared with those in the GP group. Except a strong connection with vascular anatomical complexity of the patients, these disparities also might be related with surgical skills. Therefore, care should be taken to identify these anatomical variants and to carefully dissect lymphatic fatty tissue at the inferior border of the pancreas.

## CONCLUSION

We have described 6 types of confluence of the RGEV and summarized the division points of the RGEV during laparoscopic gastrectomy for different drainage patterns. For types IV, V, and VI, the surgeon should carefully vascularize and divide the RGEV above its confluences during surgery.
